# Multipronged management of chronic non-healing venous leg ulcers using advanced wound dressings and patient-centered care: a case report

**DOI:** 10.1186/s13256-026-06059-y

**Published:** 2026-05-05

**Authors:** Dilip Kumar Kandar, Keshavi Killi, Debasis Chakrabarti

**Affiliations:** 1 Kandar Diabetes Centre, Tarnaka, Hyderabad, Telangana 500017 India; 2 Quorit Wound Care and Mentor, Bionest DMIHER, Wardha, Maharashtra 442107 India

**Keywords:** Leg ulcers, Diabetes management, Nutrition, Patient education, Case report

## Abstract

**Background:**

Venous leg ulcers are chronic wounds that can persist for years and severely affect quality of life, especially when complicated by infection and metabolic disturbances. This case is atypical, as it documents the complete healing of long-standing, bilateral venous leg ulcers that persisted for nearly a decade and became complicated by the onset of type 2 diabetes and Pseudomonas aeruginosa infection. The report highlights a hypothesis that prolonged inflammation in chronic wounds may contribute to metabolic dysregulation, while also demonstrating how an integrated outpatient multipronged management strategy can achieve complete healing without hospitalization.

**Case presentation:**

A 65-year-old South Asian man with a 10-year history of non-healing venous leg ulcers on both legs presented with foul-smelling discharge, thickened wound edges and social isolation due to odor. He had been diagnosed with type 2 diabetes 5 years ago and hypertension for 10 years. On examination, he had a large left-leg ulcer measuring 13 × 10 cm and two right-leg ulcers measuring 11 × 11 cm and 7 × 7 cm. Laboratory tests showed elevated inflammatory markers (C-reactive protein 248 mg/L, erythrocyte sedimentation rate 102 mm/h), mild anemia and wound swab culture/sensitivity positive for *Pseudomonas aeruginosa*, sensitive to meropenem and amikacin. Management included debridement, antibiotic therapy and application of a bioactive collagen–nanosilver gel dressing with a four-layer compression bandage. Nutritional counseling focused on high-protein meals, vitamin D and zinc supplementation, and adequate hydration. Regular family counseling sessions and follow-up visits were conducted to ensure adherence, leading to complete healing of all ulcers with restoration of mobility and self-care ability. No recurrence of ulcers was observed during follow-up.

**Conclusions:**

This case demonstrates that a multipronged outpatient approach combining targeted antimicrobial therapy, bioactive collagen–nanosilver wound dressings, nutritional optimization, and patient and caregiver education can result in full healing of chronic, infected venous ulcers while reducing the need for inpatient care. It also raises the hypothesis that chronic systemic inflammation from long-standing ulcers may predispose to metabolic dysfunction. Further prospective studies are needed to explore this potential link and to evaluate the reproducibility and cost-effectiveness of such multidisciplinary interventions in chronic wound management.

## Background

Venous leg ulcers (VLUs) represent 70–90% of all chronic leg ulcers [1], affecting quality of life and imposing a substantial socioeconomic burden [2]. The prevalence of VLUs is reported to be around 1.08% [2] globally, while few epidemiological reported prevalence of VLU’s as 4.5/1000 and incidence of acute wounds as 10.5/1000 population in India [3]. In India, their prevalence is estimated at 4.5 per 1000 population, with venous etiology being a primary cause, often compounded by malnutrition, pressure ulcers, trauma and infectious diseases like tuberculosis, leprosy and filariasis [3]. Despite advancements in wound care, VLUs frequently persist due to factors such as patient non-compliance, poor glycemic control, social stigma and inadequate nutrition, leading to poor quality of life.

Healing of VLU’s is always an energy-intensive process, necessitating the need of several macronutrients, fluids and micronutrients to ensure proper healing. The VLU’s especially the malodorous and exuding type among patients, also lead them toward social isolation further compounding the already existing nature of ulcer and nutritional deficiency. The nutritional deficit along with psychological issues should be addressed in a multidimensional way to ensure the integrity of healing process in VLU’s.

This case report highlights a patient's experience with venous leg ulcers and the obstacles that hindered proper wound healing. It demonstrates that a patient-focused, interdisciplinary approach can significantly improve outcomes, even in the most challenging cases. This case is particularly noteworthy due to the presentation with long-standing, bilateral venous leg ulcers that had persisted for 10 years, later complicated by the development of type 2 diabetes over the preceding 5 years. The co-occurrence of chronic, non-healing ulcers and subsequent diabetes raises a hypothesis that sustained systemic inflammation from chronic wounds may have contributed to metabolic dysregulation and the onset of diabetes. This case, therefore, highlights an atypical clinical course and provides an opportunity to explore this potential link in future research.

## Case presentation

A 65-year-old South Asian man presented with non-healing ulcers in both legs (since 10 years), along with persistent leg swelling, foul-smelling discharge and intermittent episodes of fever. For the past decade, these wounds had not only caused him physical pain but also emotional distress, impacting his ability to work. A written informed consent was obtained from the patient to record the progress of the wound and also to use the data, as desired by the treating team. The case was followed during July 2024–November 2024.

The medical history revealed that he was known hypertensive and diabetic (Type 2), since 10 and 5 years, respectively, with long-standing varicose vein ulcers. The patient had been managing with medications (metformin-500 mg, glimepiride-1 mg, amlodipine-5 mg and metoprolol-50 mg). According to the patient, a previous laser surgery for varicose veins had only worsened his condition.

The patient was an uneducated barber and the occupation, demanded long-standing hours (for 30 years), and hence never indulged in any physical activity. The patient stopped smoking 10 years ago, and consumed alcohol occasionally. No significant family history was reported.

Diet history of the patient indicated that the patient was consuming only semolina upma or wheat roti with very little curry and/or little curd, due to the fear that diabetes might shoot up and hence reduced the portion sizes of food. The family could not afford consuming fruits or meat due to poor financial status. Intake of pulses and rice was stopped due to fear of increased infection. No snacks were consumed in between two major meals.

Due to ignorance, the patient was cleaning the ulcer with local over-the-counter ointment. He had visited a local clinic a couple of years ago, where a bandage was provided along with ointment. Later, because of the foul odor emanating from the VLU, he discontinued clinic visits as he did not want to expose the sticky pus and odor in the clinic. The persistent foul odor and open wounds also led family members to advise him to remain in an isolated room, which further contributed to his desolation.

On general examination, vitals were stable, and presented with mild pallor, under nourishment (BMI-20.8), weight loss (10–12 kg in past year). Local examination indicated a large 13 × 10 cm ulcer on the medial part extending onto dorsum of left leg, characterized by purulent discharge and thickened edges, and another two ulcers of 11 × 11 cm on the medial part of right leg and a smaller 7 × 7 cm ulcer on the posterior third of right leg, covered in a wet bandage emanating foul smell and greenish discoloration. Investigations revealed the following findings (Table 1).

**Table 1:** Findings of the investigations

Parameter	Result	Reference range
WBC count	13,800/µL	4,000–11,000/µL
Hemoglobin (Hb)	9.9 g/dL	12–16 g/dL
Random blood sugar (RBS)	167 mg/dL	< 200 mg/dL
HbA1c	6.1%	< 7%
Serum creatinine	1.3 mg/dL	0.6–1.2 mg/dL
C-reactive protein (CRP)	248 mg/L	< 5 mg/L
ESR	102 mm/hour	< 20 mm/hour
HIV and HBsAg	Non-reactive	–
Liver function test	Normal	–
Vibration perception	Loss of sensation	–
ABI (ankle–brachial index)	Right 0.89, left 0.73	–
Culture and sensitivity	*Pseudomonas aeruginosa*, sensitive to meropenem and amikacin	–

*WBC* white blood count, *HbA1c* glycated haemoglobin, *ESR* erythrocyte sedimentation rate, *HIV* human immunodeficiency virus, *HBsAg* Hepatitis B surface Antigen

The patient was diagnosed with Type 2 diabetes, infected venous ulcers with peripheral arterial disease (PAD). Based on ABI measurements, the patient was advised to undergo further evaluation and vascular surgery opinion. However, he declined additional investigations, expressing emotional and physical fatigue from managing chronic ulcers for over 10 years. Despite elevated inflammatory markers, there was no exposed bone or positive probe-to-bone test, lowering the immediate clinical suspicion for osteomyelitis. MRI was planned if clinical progress stalled or if suspicion for underlying bone involvement increased. A stepwise, multidisciplinary, response-guided management plan was adopted in accordance with guidelines [4, 5]. Poor diet pattern, low nutrient intake, ignorance of patient and family members and social isolation due to stigma compounded the progression of VLU.

### Therapeutic management plan

A multipronged holistic approach was adapted to ensure patients care through management of VLU, infection, wound care, nutritional support and family support. As the patient was unable to bear the cost of therapy, the treatment (medications, wound care kit, nutritional supplements) was supported by a trust*.* The chronological course of management is summarized in Table 2.

**Table 2:** Timeline of patient's journey with chronic venous leg ulcers from onset to recovery

Timeline	Summary of events
T = −10 years (2014)	Onset of symptoms; lifestyle change—smoking cessation
T = −7 years (2017)	Symptoms exacerbated by occupation; initial conservative measures
T = −5 years (2019)	Diagnosis of Type 2 diabetes mellitus; continued progression of ulcers over legs
T = −3 years (2021)	Worsening symptoms; attempted intervention—underwent a laser procedure (details unknown)
T = −2 years (2022)	Development of foul-smelling discharge; family advised limiting social interactions due to bad odor
T = −1 year (2023)	Emotional distress and loss of interest in self-care
T = 0 (05/07/2024) Visit 1/Day 1	Initial out-patient department evaluation: clinical history, photodocumentation, clinical wound measurement; baseline investigations (Table 1); surgical debridement performed; papain–urea ointment applied; sterile dressing + four-layer bandage; wound swab taken for culture and sensitivity; multidisciplinary plan started (wound care, nutrition, counselling)
T = + 3 days (08/07/2024) Visit 2/Day 4	Dressing change; wound cleaning with normal saline, pH-balanced soap, demineralized water; 4-layer bandage continued; empirical broad-spectrum antibiotics started. Family and patient counselling
T = + 7 days (12/07/2024) Visit 3/Day 8	Repeat debridement including scrubbing wound edges and dressing; mild active bleeding observed from wound bed. Culture and sensitivity result available: *Pseudomonas aeruginosa*. Initiated targeted antibiotic therapy with meropenem 1 g intravenous BD for 10 days. Nutrition and diet counselling
T = + 14 days (19/07/2024) Visit 4/Day 15	Continued local wound care and dressing as needed; early healthy granulation tissue noted. Nutritional support and supplements (high-protein diet + vitamin C + zinc) started
T = + 17 days (22/07/2024) Visit 5/Day 18	As per clinical judgment and antibiotic sensitivity, meropenem course completed and amikacin injections started for 10 days. Continued dressings and nutrition support
T = + 29 days (03/08/2024) Visit 6/Day 30	Marked reduction in wound area; dressing interval gradually extended; nutrition and counselling ongoing
T = + 40 days (14/08/2024) Visit 7/Day 41	Left leg ulcer completely healed; right-leg ulcer markedly reduced/nearing epithelialization; recurrence-prevention education and counselling reinforced
T = + 6 to 14 weeks Aug to Oct 2024	Dressings continued as needed until complete healing; nutritional regimen continued for a recommended 3 months; ongoing counselling for compliance and lifestyle modification
T = + 131 days 13/11/2024 Day 132	Follow-up visit showed complete healing of all ulcers; no recurrence or new ulceration; patient and family expressed satisfaction with complete healing outcomes

### Advanced wound care

**Fig. 1:**
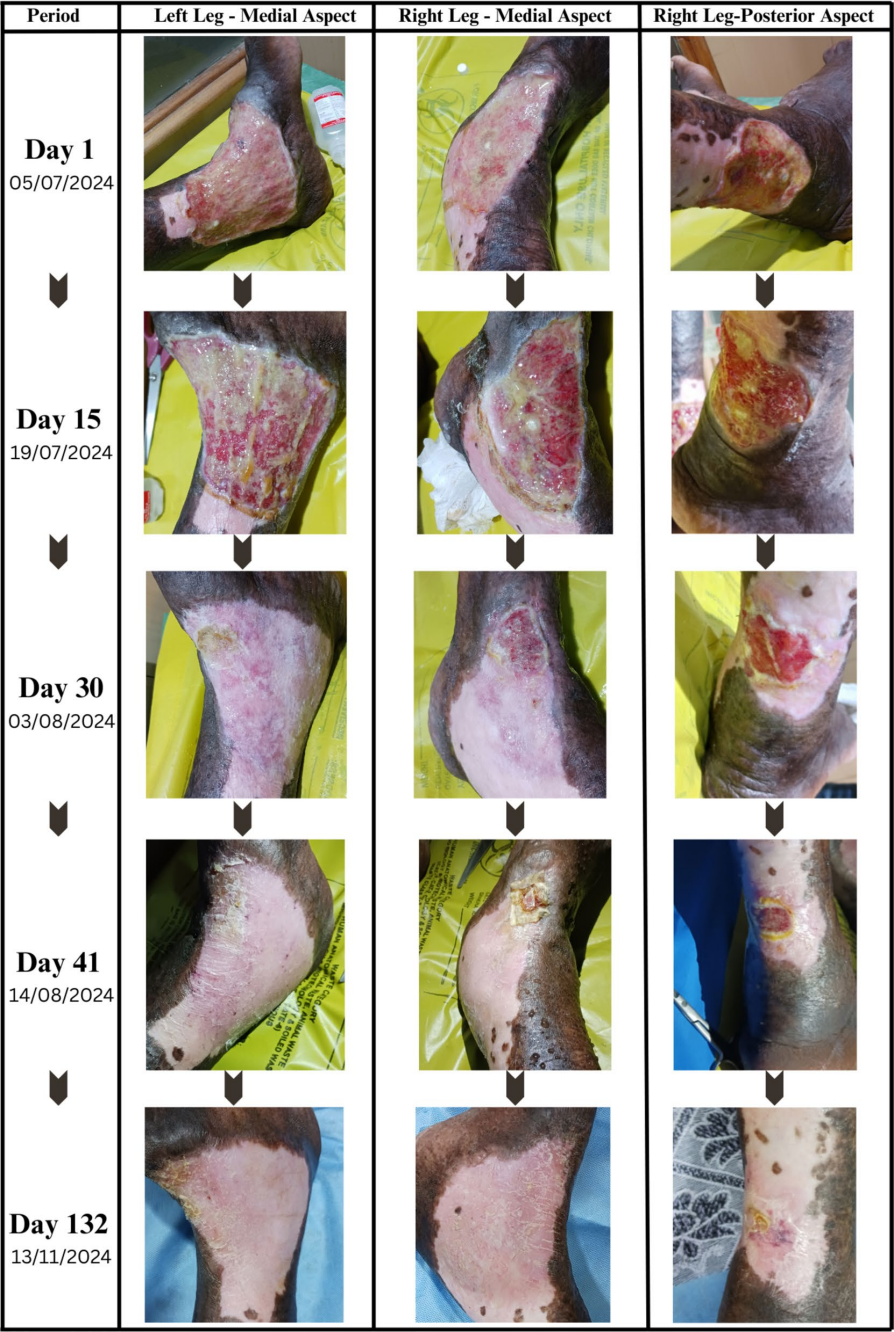
Healing of leg ulcers

- On day 1, ulcers were carefully cleaned with povidone iodine and betadine, debrided, and papain-urea ointment was applied while awaiting for the culture and sensitivity results.
- On day 3, lesser slough was noted but with profuse discharge and foul odor. The dressing was continued at the clinic with a 4-layer bandage, incorporating advanced wound dressings. These included natural bovine nanosilver collagen gel [6] enriched with Vitamin C, hydrocolloidal hydrophilic absorbable fibrous collagen membrane and non-adhesive protective dressings.
- Thorough cleaning was continued with normal saline, pH-balanced soap and demineralized water and debridement of infected tissue was continued along with scrubbing of all the wound edges on day 9. Superficial blood circulation was noticed, along with active mild bleeding from bed of the wound.
- The wound care kit, composed of bioactive dressing materials, optimized the healing process, while reducing the frequency of dressing changes. This disrupted bacterial activity and biofilm formation, aided in infection control, stimulated angiogenesis by targeting the hypoxic environment and promoted new blood vessel formation. Absorbable components regulated moisture levels while also supporting tissue regeneration and strengthening collagen integrity to enhance wound recovery. This led to direct cost savings and further indirect savings by minimizing hospital visits and enabling outpatient treatment without the need for admission.
- The patient's progress was monitored across 19 weeks over a span of 132 days. Figure 1 illustrates the significant improvements observed during the visits, culminating in recovery of the leg ulcers. Throughout this period, the effectiveness of dressing approach was evaluated, ensuring it supported healing while also helping to ease the patient's financial burden.

### Glycemic control and inflammation management

Blood glucose levels were closely monitored, while the patient's diabetes and hypertension medications were reviewed and adjusted, with doses titrated and the regimen modified to meet the patient's specific needs throughout the course of treatment. FDC of Bromelain 180 mg, Trypsin 96 mg, and Rutoside 200 mg were prescribed to reduce edema, pain and inflammation.

### Antibiotic therapy

Empirical broad-spectrum antibiotics (ofloxacin 200 mg BD and ornidazole 500 mg BD) were initially prescribed for 3 days, while awaiting microbiology results. Following culture and sensitivity results, which identified *Pseudomonas aeruginosa* sensitive to meropenem and amikacin. Empirical therapy was discontinued, and meropenem 1 g IV (intravenous) BD was administered for 10 days in an ambulatory wound-care setting starting on Day 4, followed by Amikacin 500 mg IM (intramuscular) OD for the subsequent 10 days. This was in accordance with the recent guidelines, regarding prevention of antibiotic resistance through targeted, evidence-based therapy [5].

### Nutritional support

FDC (antioxidants) of L-carnitine, L-tartrate, L-arginine, zinc, lycopene and ubidecarenone; Vit.D3-60,000 IU/week, methylcobalamin-1500mcgOD and high biological value protein 15-20gm/day were prescribed.

### Diet counseling

The clinical nutritionist worked with the patient closely to improve diet pattern, emphasizing high-quality protein; increased consumption of fruits, vegetables, meat, pulses, and improved hydration all of which helped restore his strength and confidence. The diet pattern continued for 3 months, with complete family support. Counseling session to patient and family members improved clarification on portion control/myths and fear factors which aided in healing of ulcers.

### Outcome

After a tough start, with no signs of improvement in the first few days, the treatment began to show effects. Following thorough debridement and mild bleeding was observed, which indicated the beginning of healing process. With tailored nutritional support, the patient gradually regained his strength, allowing him to participate more actively in self-care. This case demonstrates the importance of treating chronic ulcers through a multidisciplinary lens. By addressing not just the physical wounds but also the patient’s overall well-being, including nutrition, emotional support and fostered family care created an environment conducive to healing.

### Objective wound outcome measurements

Objective wound outcome data were recorded to quantify healing progress. Wound dimensions (maximum length and perpendicular width in cm) were measured at each follow-up. Wound area was calculated assuming an elliptical shape using the formula area = π × (length/2) × (width/2), and the percent area reduction was computed as (baseline area − follow-up area)/baseline area × 100. Measurements were based on clinical recordings during dressing changes, photographs lacked scale bars, so some values were estimated from clinical notes. The healing progress over 132 days is summarized in Table 3.

**Table 3:** Wound size and area reduction at serial follow-ups

Timepoint (date)	Left leg—medial + dorsum	Area (cm^2^)	% Reduction	Right leg—medial	Area (cm^2^)	% Reduction	Right leg—posterior	Area (cm^2^)	% Reduction
Day 1 05/07/2024	13 × 10 cm	102.0	0	11 × 11 cm	95.0	0	7 × 7 cm	38.5	0
Day 15 19/07/2024	11 × 8 cm	69.0	32.4	9 × 9 cm	63.6	33.1	6 × 6 cm	28.3	26.5
Day 30 03/08/2024	2 × 3 cm	4.7	95.4	3 × 4 cm	9.4	90.1	4.5 × 4.5 cm	15.9	58.7
Day 41 14/08/2024	Healed (0 cm^2^)	0.0	100	1.5 × 1 cm	1.2	98.7	2 × 2.5 cm	3.9	89.9
Day 132 13/11/2024	Healed (0 cm^2^)	0.0	100	Healed (0 cm^2^)	0.0	100	Healed (0 cm^2^)	0.0	100

## Discussion

The present case study demonstrates that the patient who was not a diabetic during the onset of VLU, subsequently developed type 2 diabetes during the progression of VLU’s, suggesting that elevated inflammatory markers such as CRP may have a role in the development of type 2 diabetes. The relationship between the two inflammatory cytokines IL-6 and CRP and type 2 diabetes risk were explored and a significant association of elevated levels of these cytokines were reported to be involved in the etiology and pathogenesis of incident diabetes [7, 8]. While we hypothesize a similar mechanism in this case, further research, including larger case series and prospective studies, is needed to investigate this potential link and better understand the role of chronic inflammation from VLUs in the development of metabolic dysregulation and diabetes.

Poor wound healing in long-term VLU’s is mostly aggravated by bacterial colonization and superimposed bacterial infections which improve with intravenous and oral antibiotics, thereby improving the recovery process. An increased healing rate with antibiotics in VLU’s was observed at 4–6 weeks compared to placebo [9].

Optimal wound healing requires tight glycemic control to restore cellular functions, reduce inflammation, and improve outcomes [10], coupled with adequate nutrients to support angiogenesis, epithelial growth, collagen synthesis, and wound strength [11] especially in patients with diabetes.

Nutrition was another overlooked factor until multidisciplinary care was implemented. Macronutrient repletion (30–35 kcal/kg/day, 1 g/kg protein, and 25 g/day fats) was tailored to address catabolism and support collagen synthesis [12]. Amino acids like arginine and glutamine were emphasized for their roles in immune regulation and tissue repair [12]. Micronutrient support was also essential. Vitamin D supplementation addressed immune modulation deficits and potential antimicrobial peptide dysfunction [13], while zinc supported epithelialization and reduced oxidative stress [14].

The patient in our case was educated thoroughly about optimum hydration, which was ignored for a very long time as per the patient history. Hydration, an often underplayed nutrient that aids in micronutrient transportation and elimination of metabolic waste from the body, also has a significant role in the wound-healing process, beyond mere caloric needs. Optimal fluid intake ensures tissue perfusion, maintains skin turgor and facilitates oxygen delivery, which are key components in the healing cascade [11].

**Fig. 2:**
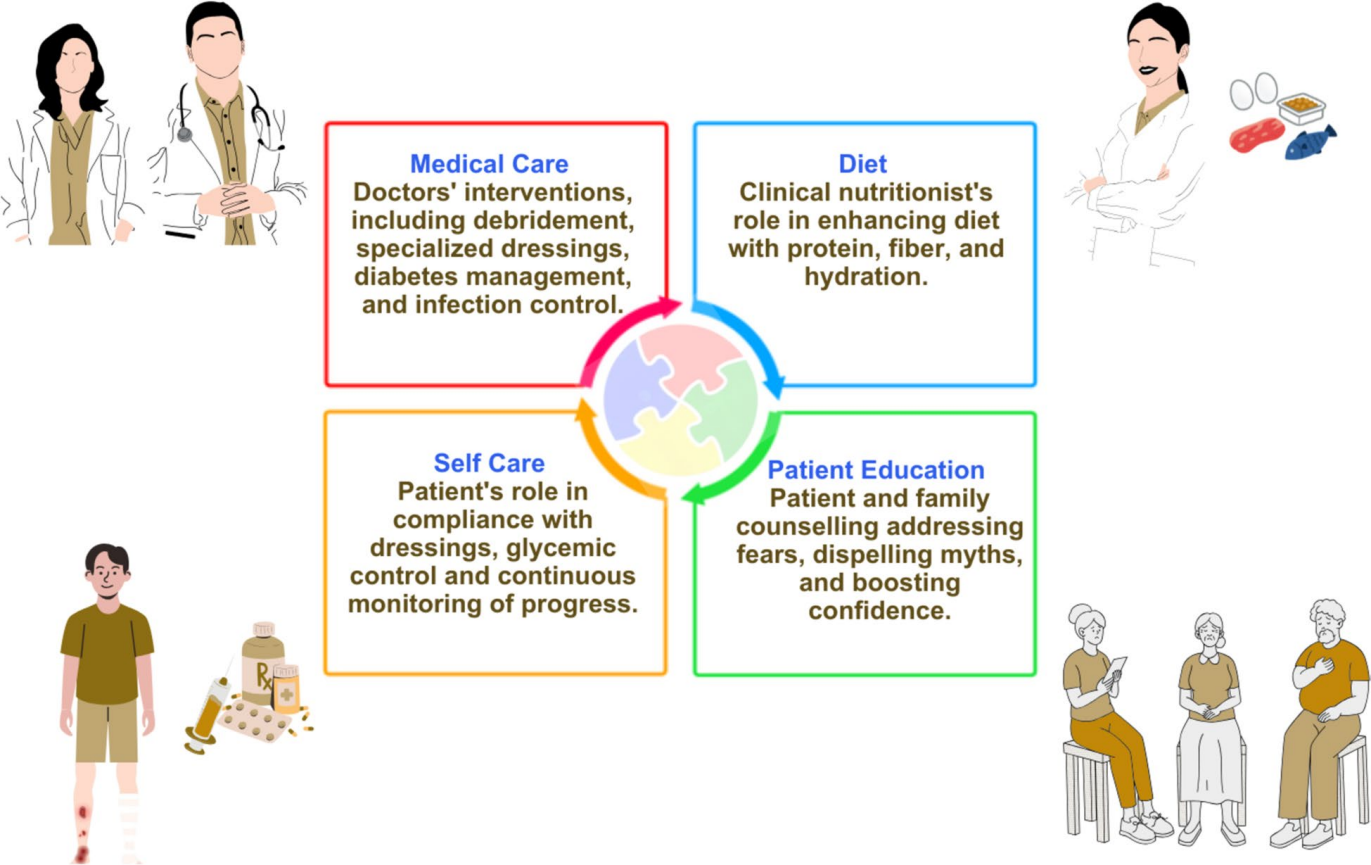
Dimensions of multipronged management of chronic non-healing venous leg ulcers

A multipronged approach in managing chronic VLU’s should always include comprehensive education for patients and caregivers, to ensure appropriate healing ecosystem as well as compliance toward appropriate care (Fig. 2). In our case study, consistent monitoring by team of doctors, podiatrician, nutrition counseling, patient education created a conducive environment for addressing the healing process of chronic VLU’s which never saw regression in 10 years time from the onset. The involvement of patient and patient’s family with a multidimensional approach such as education, supervision, control and constant monitoring of infection, along with nutrition care such as identification of malnutrition, correction of deficits and early referral to dieticians always is crucial as it improves the outcome of VLU treatment regimen [15].

### Limitations

A limitation of this patient’s history is the incomplete documentation of the patient’s prior therapies. Old medical records were missing due to frequent changes of houses, as mentioned by the patient and thus details of previous surgical, compression, antibiotic or vascular treatments could not be fully verified. The information presented is therefore based primarily on the patient’s recall and available clinical findings at the time of presentation. Another limitation is that Fig. 1 did not include a physical scale. Wound dimensions were measured clinically and documented in the clinical notes. All dimensions represent maximum measured values and may slightly overestimate actual wound size due to irregular margins. Furthermore, this case report includes only qualitative observations of quality-of-life (QoL) improvement and perceived cost savings. No validated QoL scale was administered, and no formal cost analysis was performed. The references to improved QoL and reduced costs are therefore subjective, based on patient and caregiver feedback and clinical observations of fewer dressing changes and outpatient-based care. Future case series should include validated QoL instruments and cost savings data to substantiate these findings objectively.

## Conclusion

A comprehensive approach to care, focusing on diabetes management, improved nutrition and meticulous wound care, provided by an interdisciplinary team including doctors, dietitians, patient educators and family members, led to recovery from decade-long chronic venous ulcers through proper adherence to therapy, a balanced diet and social well-being. This case reinforces the value of holistic treatment in tackling complex health issues and inspires hope for others facing similar challenges.

## Data Availability

Entire data are available in the Kandar Diabetes Centre*,* Hyderabad, India, and the reports are available in the laboratory attached to the clinic where the case study was recorded.
